# Two-year treatment effects of the common elements treatment approach (CETA) for reducing intimate partner violence and unhealthy alcohol use in Zambia

**DOI:** 10.1017/gmh.2021.2

**Published:** 2021-02-19

**Authors:** Jeremy C. Kane, Nancy Glass, Paul A. Bolton, John Mayeya, Ravi Paul, Mwamba Mwenge, Laura K. Murray

**Affiliations:** 1 Department of Epidemiology, Columbia University Mailman School of Public Health, New York City, NY, USA; 2 Department of Mental Health, Johns Hopkins Bloomberg School of Public Health, Baltimore, MD, USA; 3 Johns Hopkins University School of Nursing, Baltimore, MD, USA; 4 Department of International Health, Johns Hopkins Bloomberg School of Public Health, Baltimore, MD, USA; 5 Zambia Ministry of Health, Lusaka, Zambia; 6 Department of Psychiatry, University of Zambia School of Medicine, Lusaka, Zambia; 7 Centre for Infectious Disease Research in Zambia, Lusaka, Zambia

**Keywords:** Intimate partner violence, randomized controlled trial, transdiagnostic therapy, unhealthy alcohol use, Zambia

## Abstract

**Background:**

Intimate partner violence (IPV) and unhealthy alcohol use are common yet often unaddressed public health problems in low- and middle-income countries. In a randomized trial, we found that the common elements treatment approach (CETA), a multi-problem, flexible, transdiagnostic intervention, was effective in reducing IPV and unhealthy alcohol use among couples in Zambia at a 12-month post-baseline assessment. In this follow-up study, we investigated whether treatment effects were sustained among CETA participants at 24-months post-baseline.

**Methods:**

Participants were heterosexual couples in Zambia in which the woman reported IPV perpetrated by the male partner and in which the male had hazardous alcohol use. Couples were randomized to CETA or treatment as usual plus safety checks. Measures were the Severity of Violence Against Women Scale (SVAWS) and the Alcohol Use Disorders Identification Test (AUDIT). The trial was stopped early upon recommendation by the trial's DSMB due to CETA's effectiveness following the 12-month assessment. Control participants exited the study and were offered CETA. This brief report presents data from an additional follow-up assessment conducted among original CETA participants at a 24-month visit.

**Results:**

There were no meaningful changes in SVAWS or AUDIT scores between 12- and 24-months. The within-group treatment effect for SVAWS from baseline to 24-months was *d =* 1.37 (*p* < 0.0001) and AUDIT was *d =* 0.85 (*p* < 0.0001).

**Conclusions:**

The lack of change in levels of IPV and unhealthy alcohol use between the 12- and 24-month post-baseline timepoints suggests that treatment gains were sustained among participants who received CETA for at least two years from intervention commencement.

## Introduction

Intimate partner violence (IPV) is a substantial contributor to disease burden, increased health expenditures, and economic costs in low- and middle-income countries (LMIC) such as Zambia (Zambia Central Statistical Office, 2014). Risk for women experiencing IPV is increased when their male partners have unhealthy alcohol use (Foran and O'Leary, 2008). Unhealthy alcohol use is a highly prevalent problem in Zambia (Vinikoor *et al*., 2020), yet, there are few available evidence-based interventions for IPV and unhealthy alcohol use in LMIC and low resource settings. Most IPV intervention studies evaluate structural and economic-focused primary prevention interventions (Bourey *et al*., 2015), not secondary or tertiary prevention approaches that are designed to address individual-level risk factors such as alcohol use and mental health problems, and few IPV, alcohol, and mental health studies in LMIC have long-term follow-up outcomes assessments.

In a randomized-controlled trial, we found that the common elements treatment approach (CETA), an evidence-based transdiagnostic psychotherapy, was effective in reducing both IPV and unhealthy alcohol use compared to treatment as usual plus safety check (TAU-Plus) among heterosexual adult couples in Zambia at a post-treatment follow-up and at a 12-month post-baseline assessment. The trial's data and safety monitoring board (DSMB) recommended that the trial be stopped early due to effectiveness and CETA was provided to TAU-Plus participants following the 12-month analysis (Murray *et al*., 2020). We followed the original CETA participants through to a 24-month post-baseline assessment and this brief report presents findings on the two-year CETA treatment effects.

## Methods

### Study design and sample

The original trial methods are published in a protocol paper (Kane *et al*., 2017), the primary outcomes paper (Murray *et al*., 2020), and registered on ClincialTrials.gov (NCT02790827). Ethical approval was obtained from the Johns Hopkins Bloomberg School of Public Health IRB and the University of Zambia Biomedical Research Ethics Committee.

Briefly, 248 couples were recruited by local lay counselors trained in CETA. Counselors went door-to-door in their communities to inform couples about the study. Interested couples were referred to the research team, consented, and completed a screener housed on an audio computer-assisted self-interviewing (ACASI) laptop. The man and the woman in the couple were screened separately. Women completed the Severity of Violence Against Women Scale (SVAWS) (Marshall, 1992), a 46-item assessment of experienced IPV severity that includes subscales of threatened violence and physical/sexual violence, and the Alcohol Use Disorders Identification Test (AUDIT) (Saunders *et al*., 1993), a 10-item measure of unhealthy alcohol use. Two versions of the AUDIT were administered: one in which the woman's own drinking was evaluated (self-reported AUDIT) and one in which she was asked to report on her male partner's drinking (partner-reported AUDIT). Men completed a self- and partner-reported AUDIT but not the SVAWS. Couples were eligible if the woman reported at least moderate levels of IPV (⩾38 on the SVAWS physical/sexual violence subscale) and the man had hazardous alcohol use as evidenced by a score of ⩾8 on the woman's partner-reported AUDIT or on the man's self-reported AUDIT. Eligible couples were randomized on a 1:1 basis to CETA or TAU-Plus. Men and women in couples randomized to CETA received separate CETA sessions (approximately 6–12 one-hour weekly sessions). Couples receiving TAU-Plus did not receive a formal intervention, but the study team conducted regular check-ins with these couples for ethical and safety purposes.

### Measures

Outcomes included the SVAWS (recent physical/sexual violence and threatened violence subscales administered to women only), the World Health Organization (WHO) Multi-Country on Women's Health study (World Health Organization, 2005), which includes two binary items on whether there was *any* recent physical IPV and *any* recent sexual IPV (women were asked about experiencing IPV; men were asked about perpetrating IPV), and the AUDIT (both self- and partner-reported versions administered to both men and women). Outcomes were assessed via ACASI at baseline/screening, post-treatment (approximately 3–4 months post-baseline for TAU-Plus participants), and 12-months post-baseline with a planned additional assessment at 24-months post-baseline. Following the DSMB determination to stop the trial early at 12 months, all TAU-Plus participants exited the study and were offered CETA. We continued to follow the original CETA participants and conducted a 24-month post-assessment.

### Statistical analysis

Statistical analysis for the between-group effectiveness analysis was intent-to-treat and included all enrolled participants following multiple imputation (Azur *et al*., 2011). Mixed effects models with robust standard error estimators that included fixed effects of treatment group, time, and a group by time interaction and random effects of participant and counselor ID were estimated. The original models included all study participants (CETA and TAU-Plus) and the three original timepoints (baseline, post-treatment, 12 months post-baseline). The new analysis being presented in this paper also used mixed effects models but included only CETA participants and the baseline and 24-month post-baseline assessment data (TAU-Plus participants and the post-treatment and 12-month data were excluded). The fixed effect of interest in these new models was time. Predicted 24-month means and percentages were generated from the models as were within-group Cohen's *d* effect sizes (for continuous outcomes-SVAWS and AUDIT) and relative risks (RRs; for binary outcomes from the WHO IPV measure).

Original data collection for the trial was conducted between 23 May 2016 and 16 April 2018 (date of the last 12-month assessment). Twenty-four-month follow-up assessments among original CETA participants were conducted between 23 May 2018 and 26 January 2019.

## Results

Of the 123 couples randomized to CETA, *N* = 97 (78.9%) women and *N* = 93 men (75.6%) completed a 24-month post-assessment. Table 1 shows the 24-month post-baseline assessment results for CETA participants and the reference values from baseline, post-treatment and 12 months post-baseline (Murray *et al*., 2020). For the primary study outcome, SVAWS physical/sexual violence subscale, the predicted mean at 24 month post-baseline (37.5, 95% CI 33.8–41.2) was similar and slightly lower than the mean for 12 months post-baseline (41.9, 95% CI 37.6–46.2) and the within-group effect size for change in SVAWS score from baseline to 24 months was 1.37 *(p* < 0.0001). Overall, means and risk percentages at 24 months were similar to corresponding values at 12 months for the SVAWS threatened subscale, the WHO IPV indicators, and all of the AUDIT measures and there were also significant within-group treatment effects for all outcomes.

**Table 1.** 24-month outcome results among CETA participants (*N* = 123)

	Reference values from Murray *et al*. (2020)^a^				
	Baseline	Post-treatment	12-month post-baseline	24-month post-baseline results^b^	
Continuous variable	Mean (95% CI)			Mean (95% CI)	Within-group effect size, *p* value
SVAWS physical/sexual violence scale	65.2 (62.0–68.3)	38.6 (34.5–42.8)	41.9 (37.6–46.2)	37.5 (33.8–41.2)	1.37, *p* < 0.0001
SVAWS threatened violence scale	48.7 (43.8–49.6)	27.9 (24.6–31.2)	29.7 (26.2–31.1)	28.9 (25.9–32.0)	1.34, *p* *<* 0.0001
AUDIT: Male self-report	14.9 (13.3–16.4)	5.7 (4.1–7.3)	5.7 (3.7–7.7)	5.5 (3.2–7.8)	0.85, *p* *<* 0.0001
AUDIT: Female partner-report	21.7 (19.9–23.6)	9.1 (6.9–11.2)	10.0 (7.9–12.0)	11.0 (8.6–13.3)	1.11, *p* < 0.0001
AUDIT: Female self-report	11.8 (9.9–13.6)	4.5 (2.6–6.4)	5.7 (3.7–7.8)	5.4 (4.2–6.5)	0.53, *p* < 0.0001
AUDIT: Male partner-report	9.9 (8.2–11.6)	5.7 (4.0–7.4)	6.2 (4.5–8.0)	7.6 (5.9–9.2)	0.31, *p* = 0.02
Binary variable	*N* (%) 95% CI			*N* (%) 95% CI	Risk ratio (95% CI) *p* value
Any physical violence experience: female report	98 (80%) (90–108)	43 (35%) (33–55)	47 (38%) (42–77)	36 (30%) 30–48	0.38 (0.29–0.49) *p* < 0.0001
Any physical violence perpetration: male report	98 (80%) (93–105)	31 (25%) (21–47)	47 (38%) (37–58)	46 (37%) (34–60)	0.47 (0.35–0.62) *p* < 0.0001
Any sexual violence experience: female report	101 (82%) (89–113)	43 (35%) (34–55)	44 (36%) (32–60)	41 (33%) (33–52)	0.41 (0.33–0.52) *p* < 0.0001
Any sexual violence perpetration: male report	64 (52%) (55–75)	27 (22%) (18– 38)	37 (30%) (27–52)	32 (26%) (25–41)	0.50 (0.36–0.70) *p* < 0.0001

a Reference values are from analysis described in Murray *et al*. (2020). Estimated mean values are based on predicted values of mixed effects models. For binary outcomes, *N*'s are calculated based on predicted %. All participants were included in the analysis following multiple imputation of missing data.

b 24-month results are based on updated mixed effects models that included all CETA participants following multiple imputation (control participants were not included in the model). Estimated mean values are based on predicted values of mixed effects models. For binary outcomes, N's are calculated based on predicted %. Within-group effect size is calculated as the predicted change from baseline to 24-month follow-up from the mixed effects model divided by the baseline standard deviation among CETA participants. The within-group risk ratio is the predicted change in risk from baseline to 24 month-post-treatment. Risk ratios <1 indicate a reduction in risk. The associated *p* value with the effect size/risk ratio is from the time variable in the mixed effects model.

## Discussion

The results from the primary trial analysis showed that CETA was clinically and statistically significantly superior to TAU-Plus in reducing both IPV and unhealthy alcohol use among heterosexual couples in Zambia at a one-year follow-up (Murray *et al*., 2020). The present study extends those findings to suggest that the treatment gains attributable to CETA persist for at least up to two years. To our knowledge, this is the first randomized trial in sub-Saharan Africa to demonstrate an intervention for IPV and unhealthy alcohol use with two-year sustained impacts.

In addition to the limitations described in the original trial paper (Murray *et al*., 2020), the main limitation of the present analysis is the lack of a control group at 24 months. Given that there was not a significant reduction in symptoms among control participants between post-treatment and 12 months post-baseline, it is unlikely that such a reduction would have occurred between 12 and 24 months with no additional intervention.

## Conclusions

CETA is an effective treatment for IPV and unhealthy alcohol use with average sustained effects for at least two years. Future planned analyses will investigate mediators and moderators of the treatment effect.
